# HfO_2_ Memristor-Based Flexible Radio Frequency Switches

**DOI:** 10.1021/acsnano.4c11846

**Published:** 2024-12-20

**Authors:** Shih-Chieh Chen, Yu-Tao Yang, Yun-Chien Tseng, Kun-Dong Chiou, Po-Wei Huang, Jia-Hao Chih, Hsien-Yang Liu, Tsung-Te Chou, Yang-Yu Jhang, Chien-Wei Chen, Chun-Hsiao Kuan, E Ming Ho, Chao-Hsin Chien, Chien-Nan Kuo, Yu-Ting Cheng, Der-Hsien Lien

**Affiliations:** † Institute of Electronics, National Yang Ming Chiao Tung University, Hsinchu 300, Taiwan; ‡ Institute of Pioneer Semiconductor Innovation, National Yang Ming Chiao Tung University, Hsinchu 300, Taiwan; § Strategic Technology Exploration Platform, MediaTek, San Jose, California 92054-5116, United States; ∥ Taiwan Instrument Research Institute, National Applied Research Laboratories, Hsinchu 302, Taiwan; ⊥ Department of Applied Chemistry and Institute of Molecular Science, National Yang Ming Chiao Tung University, Hsinchu 300, Taiwan; # Chang Chun Plastics Co., Ltd. Hsinchu Factory, Hsinchu 303, Taiwan

**Keywords:** RF switch, memristor, flexible electronics, 6G communication, 2.5D integration

## Abstract

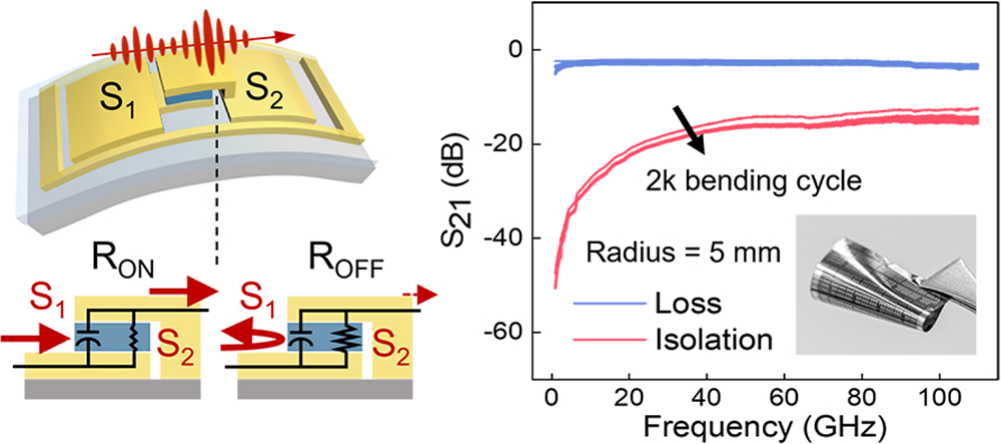

Flexible and wearable electronics are experiencing rapid growth due to the increasing demand for multifunctional, lightweight, and portable devices. However, the growing demands of interactive applications driven by the rise of AI reveal the inadequate connectivity of current connection technologies. In this work, we successfully leverage memristive technology to develop a flexible radio frequency (RF) switch, optimized for 6G-compatible communication systems and adaptable to flexible applications. The flexible RF switch demonstrates a low insertion loss (2 dB) and a cutoff frequency exceeding 840 GHz, and performance metrics are maintained after 10^6^ switching cycles and 2500 mechanical bending cycles, showing excellent reliability and robustness. Furthermore, the RF switch is fully integrable with a photolithography-processable polyimide (PSPI) substrate, enabling efficient 2.5D integration with other RF components, such as RF antennas and interconnects. This technology holds significant promise to advance 6G communications in flexible electronics, offering a scalable solution for high-speed data transmission in next-generation wearable devices.

## Introduction

Recent advancements in flexible technologies have enabled the heterogeneous integration of a diverse range of electronic and optoelectronic devices with multiplexed functionalities.^1−3^ With the incorporation of artificial intelligence (AI), flexible electronics now offers enhanced interactivity, facilitating real-time health management,^4−12^ exercise coaching^13,14^ as well as remote interventions.^15,16^ However, implementing AI into flexible electronics imposes significant demands on connectivity, requiring more efficient transmission and feedback mechanisms to handle big-data processing. While modern wearable devices already use a broad spectrum of frequencies, including Bluetooth,^17,18^ Wi-Fi,^19^ and LTE/5G,^17,20,21^ current technologies may not be sufficient for the demands of next-generation applications. The implementation of 6G, operating in the sub-7–24 GHz range and frequencies above 100 GHz, is crucial for increasing connectivity and supporting multiplexed workloads.^22−27^ Although 6G technology has advanced significantly in rigid devices, applying it to flexible circuits remains challenging due to the difficulty of maintaining reliable performance under mechanical deformation. Flexible RF switches are crucial in scenarios where stable signal transmission must be maintained under dynamic deformation. While wireless components have already been applied in flexible electronics for communications,^28^ healthcare monitoring,^29^ and epidermal electronics,^30^ their stability and reliability still require improvement,^31^ especially in the 6G communication regime.^32^

To achieve 6G communications, minimizing signal losses in integrated RF systems is crucial.^33−35^ 2.5D integration offers a promising approach by colocating RF components like antennas and RF switches on a single interposer, thereby shortening signal paths and reducing parasitic effects.^36−39^ However, maintaining signal integrity under mechanical deformation remains a significant challenge. Therefore, the development of an interposer material with high processing flexibility is critical for the successful realization of 6G communication systems in flexible electronics.

In this work, we developed flexible RF switches that exhibit 6G compatibility with structural simplicity readily for 2.5D integration. This memristor-based RF switch is made of a nanometer-thick active layer, functioning as an RF switch by offering switchable resistance states for managing the transmission of RF signal. The RF switch demonstrates a low ON-state resistance of <50 Ω, corresponding to a low insertion loss of 2 dB with a cutoff frequency over 840 GHz. The thin-film structure allows the device to sustain mechanical deformation, preventing strain-induced delamination during bending. Additionally, lithography-processable polyimide substrates is employed to achieve vertical integration of antennas and RF switches, demonstrating its 2.5D integration capability with an estimated return loss below 15 dB.

## Results and Discussion

**Figure 1:**
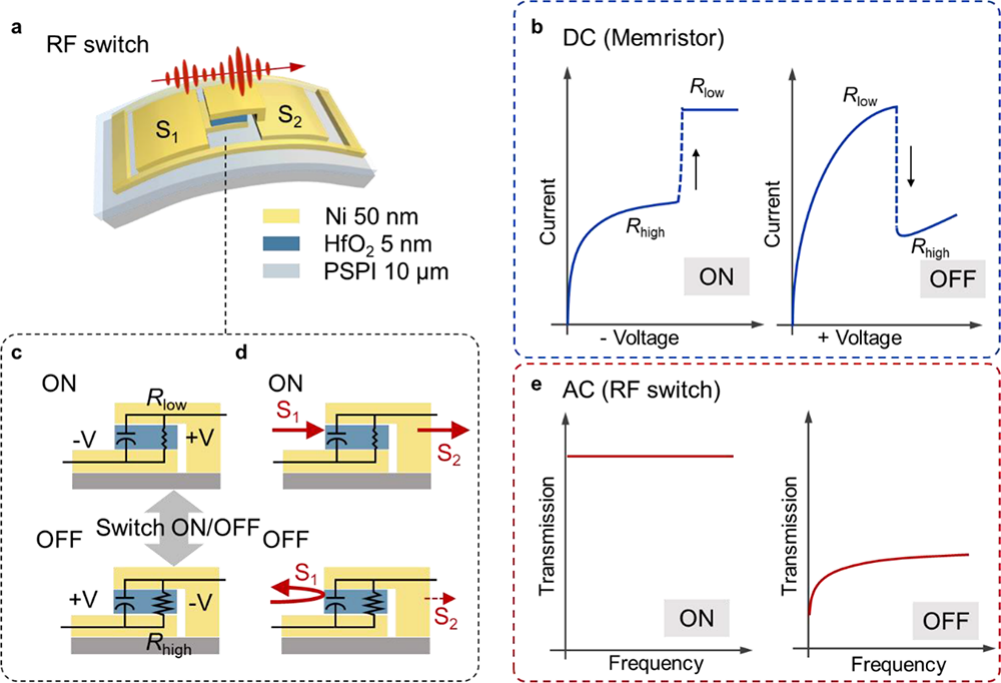
Structure and working principle of the device when it is operated as a memristor (panel enclosed by the blue-dashed rectangle) and as an RF switch (panel enclosed by the red-dashed rectangle), in DC and AC modes, respectively. (a) Schematic structure of the HfO_2_-based memristive RF switch on the PSPI/PI substrate. (b) Modulation of active layer resistance between OFF-state and ON-state by applying positive and negative DC voltages. Equivalent circuits corresponding to the ON and OFF states of the memristive RF switch when operated (c) in the DC mode and (d) in the AC mode. (e) Transmitted signal power of the switch operated in ON and OFF states.

The core of the flexible switch is a memristor with a simple metal–insulator–metal (MIM) structure that controls RF signal transmission. The memristive technologies, commonly used in nonvolatile memory, have recently expanded to applications to RF switches for mobile communication systems.^40−46^ The operational mechanism of the memristive RF switch is illustrated in Figure 1. As a memristor, the resistance of the switch is tunable between a high-resistance state (HRS) and a low-resistance state (LRS) via a direct current (DC) voltage bias, as illustrated in Figure 1b,c. The HRS and LRS, defined during the DC voltage operations, are preserved when the switch is used for RF signal transmission in an alternative current (AC) mode. The DC resistance states determine the transmission efficiency of the RF signal in high frequency. When the device is switched to the LRS, corresponding to a low-impedance state (LIS) in the AC mode, the RF signal is allowed to transmit, resulting in a larger transmitted signal power. Conversely, when the device is in its HRS state, corresponding to a high-impedance state (HIS) in the AC mode, the RF signal is reflected, leading to a low signal transmission, as depicted in Figure 1d,e.

**Figure 2:**
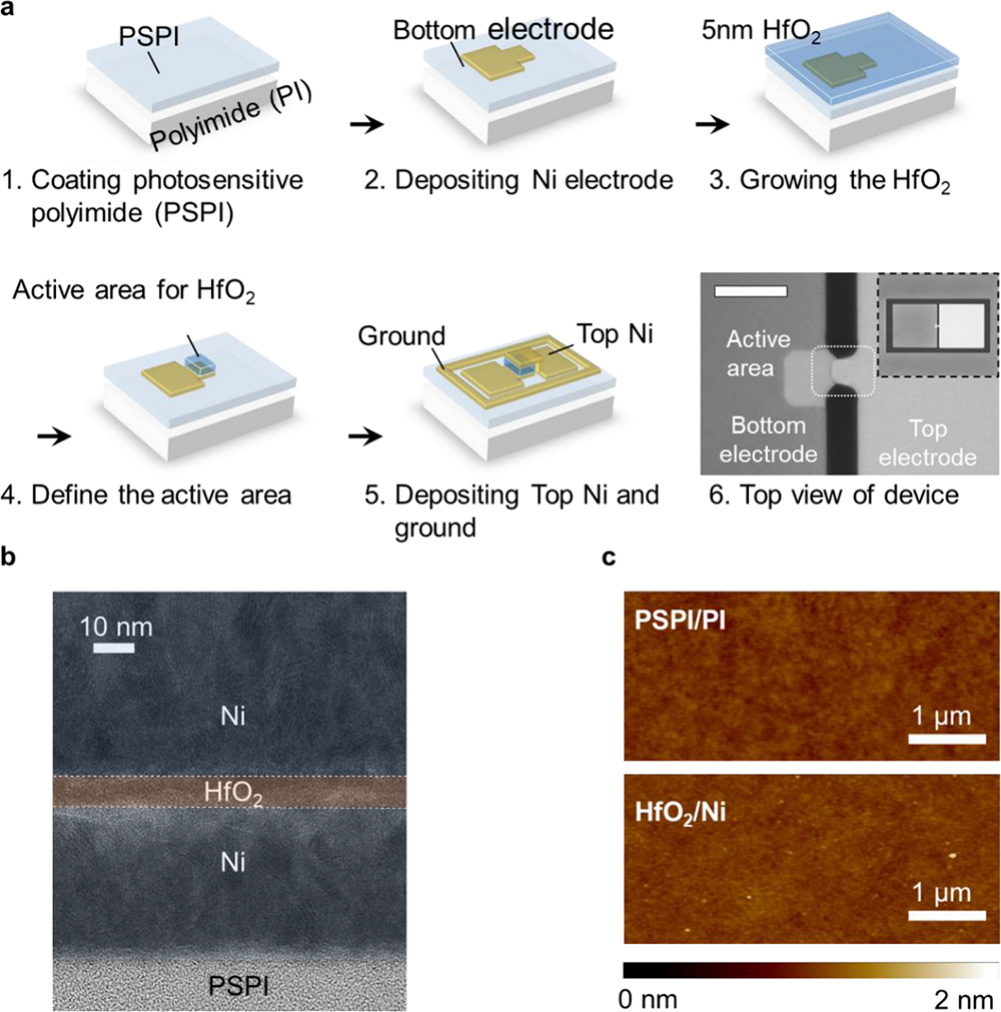
Structure and fabrication of the proposed memristive RF switches. (a) Schematic representation of the fabrication process and structure. To ensure the accuracy of measuring the insertion loss and isolation at GHz frequencies, the MIM stack is coupled with a coplanar waveguide based on a ground–signal–ground configuration to facilitate *S*-parameter characterization. A top-view optical image shows the active area of the device with a scale bar of 20 μm, while the inset provides an overview of the device (scale bar: 100 μm). (b) TEM image of the device. (c) AFM image showing the surface roughness of the flexible substrate and the integrated device.

To extend the operation frequency of the memristive RF switch, it is essential to reduce the overall impedance, specifically the resistance and capacitance of the MIM structure. A key design strategy is to decrease the thickness of the insulating layer, as the cutoff frequency of a memristor-based RF switch is inversely related to the product of its parasitic resistance and capacitance. Recently, 2D semiconductors have been demonstrated as an effective high-frequency RF switch due to their ultrathin thickness.^42,44^ 2D materials-based RF switches have shown a low ON-state resistance (*R*_on_) of 10 Ω for reduced insertion loss, which keep high impedance when they are switching off. As for flexible RF applications, however, using ultrathin insulating layers can increase the risk of current leakage under bending conditions. Therefore, an optimized thickness is required to ensure device reliability and, meanwhile, to maintain a high cutoff frequency. Based on the design criteria, a 5 nm hafnium oxide (HfO_2_) is deposited as the insulating layer by atomic layer deposition (ALD) (more details about design criteria for thickness are shown in Supporting Information Figure S1).^47,48^ As the thickness of the memristor decreases to the nanometer scale, ensuring the uniformity of each layer and managing the roughness of the flexible substrates have become critical concerns.^12^ A 10 μm photosensitive polyimide (PSPI) thin film is spin-coated onto a flexible polyimide (PI) substrate as a buffering layer (Figure 2a). The active area (5 μm × 5 μm) and structure of the RF switch are shown in the top view of the optical image in Figure 2a. The layout of the switch including the defined signal (S) and ground (G) is modified to ensure impedance matching (Figure S2). The uniformity of the multilayer devices on the flexible substrate is confirmed by transmission electron microscopy (TEM) as shown in Figure 2b. The surface roughness of the flexible substrate as well as the integrated device was determined using atomic force microscopy (AFM). AFM shows that both the PSPI/PI substrate and the deposited HfO_2_ layer on nickel metal present an ultraflat surface with a low roughness of 0.3 nm (Figure 2c), which are compatible with existing silicon-based technologies (the roughness of a commercial SiO_2_ substrate is 0.2 nm, Figure S3). To further validate the large area uniformity of the HfO_2_ layer, scanning electron microscopy (SEM) images at various magnifications (Supplementary Figure 4) demonstrate a smooth surface over the whole area for developing array devices. As a result, the device can be operated under mechanical deformation without leakage, which will be discussed later.

**Figure 3:**
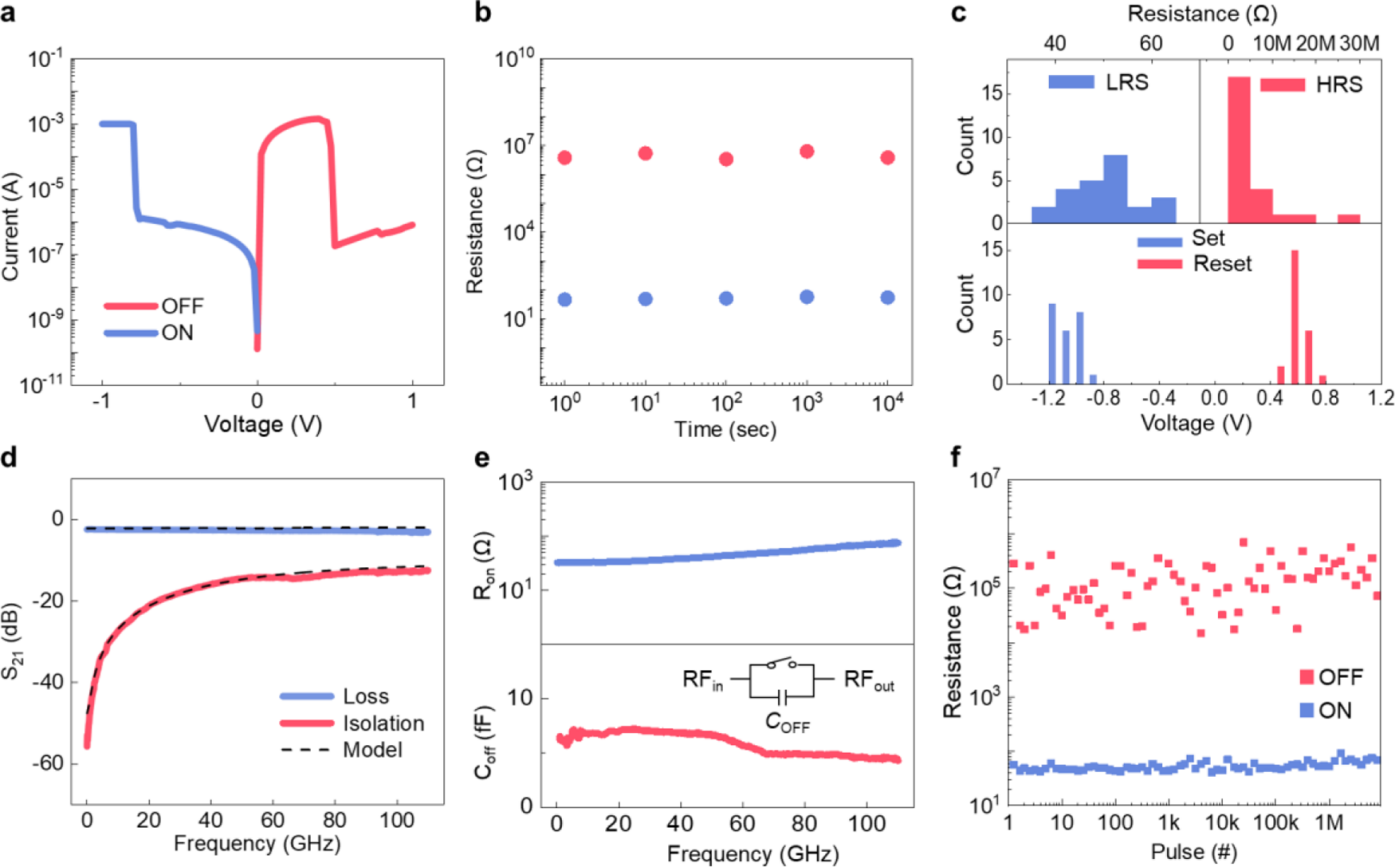
Characterization of memristive RF switches. (a) Representative *I*–*V* curve of the bipolar resistance switching behavior of the HfO_2_-memristor. (b) Retention reliability of HRS and LRS values over time, showing stable performance. (c) Distribution of HRS, LRS, and set/reset voltages across multiple devices, demonstrating consistent switching behavior. (d) *S*_21_ in both the ON-state (insertion loss) and OFF-state (isolation) of the RF switch. (e) Extracted *R*_ON_ and *C*_OFF_ of the RF switch using an equivalent circuit composed of the parallel-connected resistor and capacitor. (f) Switching characteristics of the memristive RF switch using DC pulses as the driving voltage.

Figure 3a shows the current–voltage (*I*–*V*) characteristics of the HfO_2_ memristor operated in the DC mode. The switches are initially in the LRS without the requirement of a set voltage, exhibiting forming-free characteristics. The switch remains in the LRS until a negative bias is applied to reset the memristor to an HRS (transition from LRS to HRS), with a reset voltage of ∼0.5 V (more details in Figure S5). The memristors can return to the LRS as a set voltage of ∼−1 V is applied, and the switching process is reversible. The retention characteristics of the switch are shown in Figure 3b, exhibiting that the resistance states remain stable over time. The statistical analysis in Figure 3c shows that the average values of the HRS and LRS are 4 MΩ and 53 Ω, respectively, with a ratio greater than 4 orders of magnitude.^45^ The reliable switching characteristics of HfO_2_-based memristors have been attributed to their tendency to form oxygen vacancies,^47,48^ which ensures that filaments can reliably form and remain stable over many switching cycles, making them a robust option for flexible applications.

While operating under the AC mode, the device is effectively a nonvolatile RF switch with tunable impedance states to control the transmission of RF signal. Figure 3d displays the signal transmission characteristics of the RF switch ranging from 1 to 110 GHz. As the device is in its LIS (the switch is ON), it shows a low insertion loss of 2 dB. Here, the transmission coefficient (*S*_21_) is measured as the ratio of the transmitted signal power from port S1 to port S2. As it is in its HIS (the switch is OFF), the isolation increases from 60 to 10 dB at the frequency region of 1–110 GHz. To further quantify the signal modulation capability, the parasitic resistance and capacitance of the RF switch were extracted. Figure 3e shows the extracted *R*_ON_ and the OFF-state capacitance (*C*_OFF_) of the RF switch across 5G to 6G frequency ranges. Here *R*_ON_ is derived from the low-frequency insertion loss in the LIS, reflecting the switch’s intrinsic loss, while *C*_OFF_ is derived in the HIS. The lowest *R*_ON_ for the proposed RF switch is around 32 Ω, attributed to their thin thickness. Given an average *C*_OFF_ of 5.8 fF, a cutoff frequency of 840 GHz (*f*_c_ = 1/2π*RC*) can be obtained, which covers the 6G communication range (the impact of the various substrates on RF switch performance is shown in Figures S6–S8).^42^Figure S9 shows the power handling characteristics of the RF switch, measured at a frequency of 94 GHz. Experimental data indicate that the insertion loss remains stable at varying input power levels in the ON state, demonstrating reliable performance for RF power transmission, while the OFF state exhibits a flat output power curve, indicating effective isolation of the input signal (Figure S9a,b). Figure S9c presents pulse test results at different input voltages, showing that no state change occurs until the input voltage reaches 2 V, confirming that no self-switching behavior occurs at an input power close to 10 dBm with a peak voltage (*V*_p_) of 1 V. The performance of the proposed device is comparable to current CMOS-based RF switch in terms of insertion loss and isolation at frequencies below 50 GHz and surpasses them at frequencies beyond 50 GHz. To demonstrate the endurance of the switch, a repeated pulse cycle (voltage height of 2 V and a pulse width of 200 ns) was performed to turn ON and OFF the device. The results showed a coefficient of variance (CV) of 88.32% for HRS and 18.70% for LRS. Our device shows reliable switching behavior for 10^6^ cycles and maintains a memory window of 3 orders (Figure 3f), exhibiting one of the best reliabilities among memristor-based RF switches (details of switching speed analysis in Figure S10). We have also included the performance of the device after one year in Figure S11, demonstrating that the device maintained its performance even after being stored in ambient conditions.

**Figure 4:**
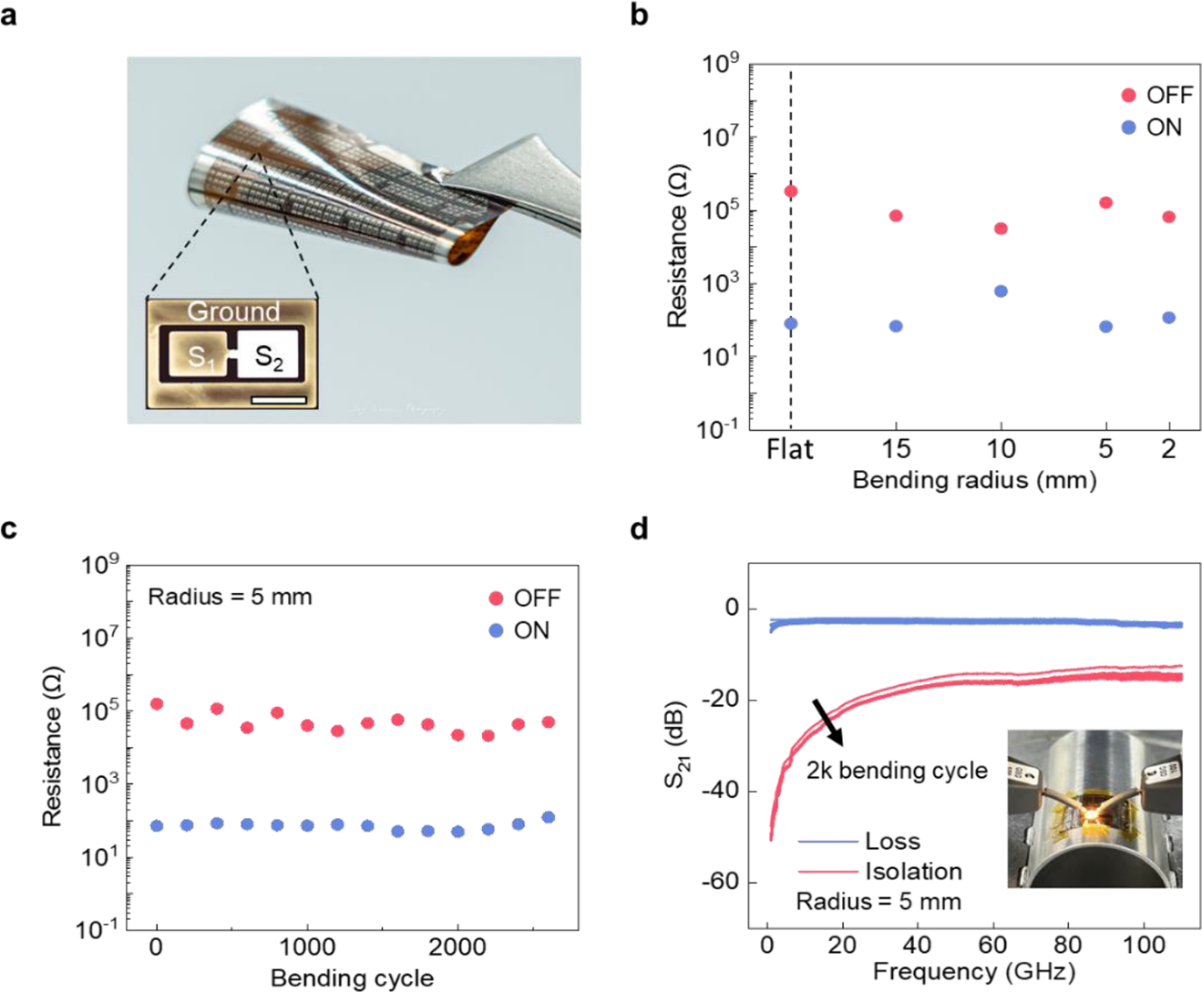
Characterization of the proposed devices for memristive and RF switching functionalities under different bending conditions. (a) Images of the flexible memristive switch. The scale bar for the inset is 100 μm. (b) HRS and LRS of the memristor measured while it was placed on curved mounts with bending radii ranging from 2 to 15 mm. (c) HRS and LRS of the memristor as a function of bending cycle with a bending radius of 5 mm. (d) High-frequency response curves after 0–2000 bending cycles show minimal change, indicating stable performance under repeated bending.

The switching performance of the flexible RF switch is then characterized under various bending conditions to demonstrate its potential for flexible applications (an image of the flexible RF switch under bending is shown in Figure 4a). The device shows consistent switching characteristics under various bending radii ranging from 15 to 2 mm (Figure 4b), and the performance of the device sustains after 2500 bending cycles (Figure 4c). When the device is bent at a radius of 5 mm and fed with an RF signal up to 110 GHz, the insertion loss remains ∼2 dB for ON-state and the isolation for OFF-state shows no significant changes (Figure 4d), demonstrating the reliability of the switch under the mechanical strain (see Figure S12 for *C*_OFF_ under mechanical bending conditions). Compared to other RF switches, our device offers both flexibility and reliability at low operating voltages (see Table S1) with a cutoff frequency of 840 GHz that holds great promise for enabling 6G communications in wearable electronics and other flexible applications.^26,27^

**Figure 5:**
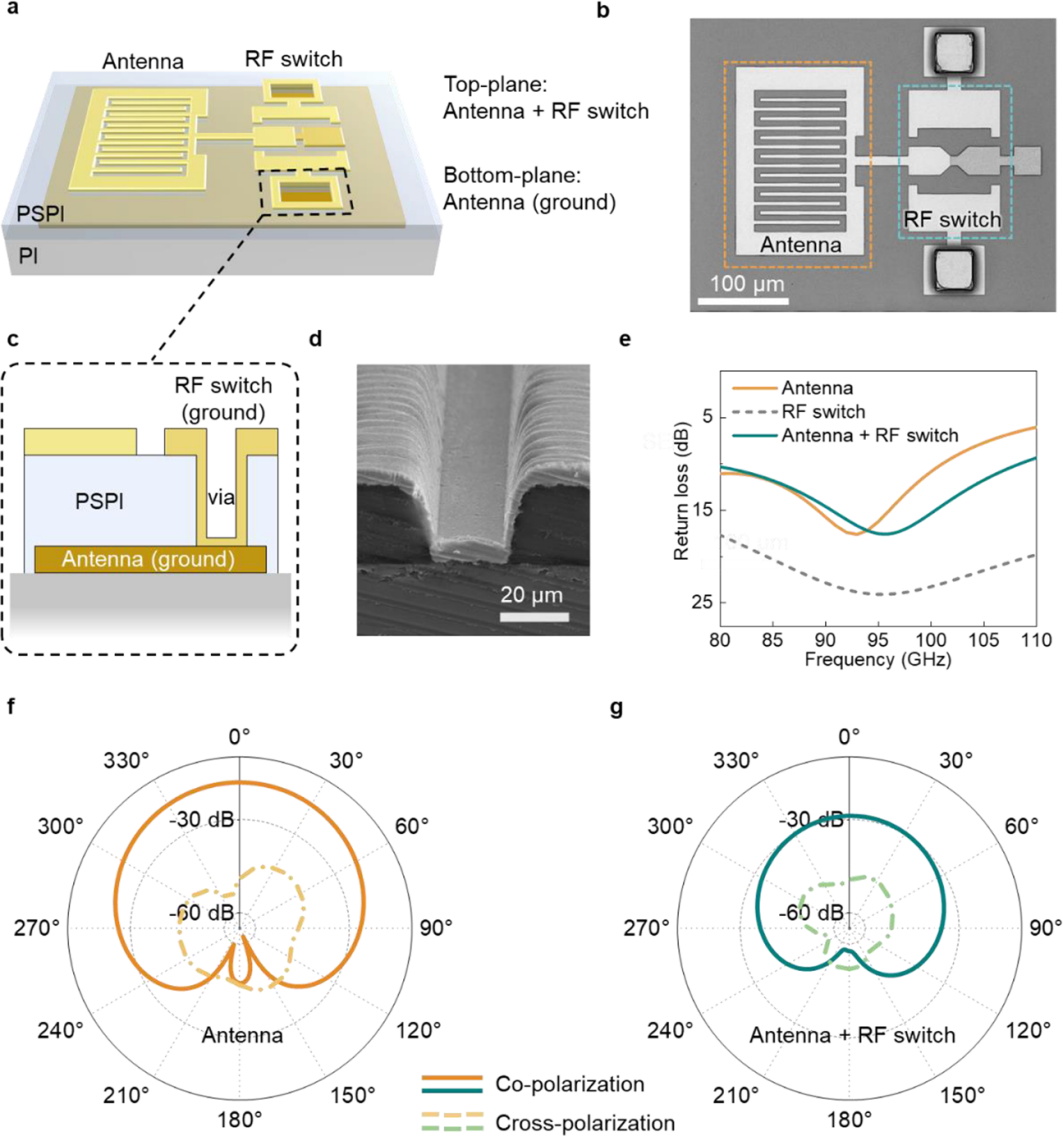
Structure and simulated characterization of the 2.5D integrated RF system. (a) 2.5D integrated system, where the RF switch and antenna are positioned on the top side of the PSPI, and the antenna ground is located under the PSPI. (b) Optical image of the 2.5D integrated RF system. (c) Illustration of the via and the interconnect. The ground for the RF switch (on the PSPI) is connected to the antenna ground (beneath the PSPI) by an interconnecting through-hole (via). (d) SEM images of the via. (e) Return Loss for the antenna, RF switch, and the 2.5D integrated system. (f, g) Radiation patterns of the standalone antenna and the 2.5D integrated system.

Here we demonstrate the 2.5D integration of the memristive RF switch on the PSPI substrates. Since PSPI is photolithography-processable, each RF component and via area can be precisely patterned, enabling the development of a complete interposer on the substrate. Figure 5a shows a diagram of the 2.5D integrated system. The RF switch and antenna were developed on the top side of the PSPI, where the ground for the RF switch (on the PSPI) and the ground for the antenna (under the PSPI) are connected through the via. The optical image of the developed system is shown in Figure 5b. Figure 5c illustrates the cross-section of the via with filled metal as the interconnect. The via area was defined by photolithography, and the via was formed by dissolving the exposed PSPI using a developer solution (2.3% TMAH). Then, the via was filled with metal using the inkjet-printed silver (details in Figure S13). The interconnect provides a low-impedance pathway that links the ground of all RF components, effectively shielding against radio frequency interference and ensuring signal integrity. Note that the entire 2.5D integrated structure remains flexible, as the components are consistent with those previously described. Figure 5d shows a scanning electron microscopy (SEM) cross-section image of the via, highlighting the excellent filling quality and high coverage uniformity achieved using the inkjet-printing technique. Figure 5e shows the return loss of the antenna, RF switch, and the integrated system (simulation details are provided in the Supporting Information), with all data in Figure 5e–g being simulated. The results indicate the antenna-RF switch integrated system maintains the signal integrity at 94 GHz with a return loss as low as 15 dB, which is the same level as compared to performance of the unintegrated antenna. Note that the return loss of the antenna combined with the RF switch is lower than that of the standalone antenna in the 80–95 GHz range, which is due to the dominant influence of the antenna on the return loss in the combined antenna-switch system (Figure S14). Figure 5f,g indicates the radiation patterns of the single antenna and the antenna combined with the RF switch. The radiation patterns demonstrate that after integrating the antenna with the RF switch, the gain and radiation performance of the antenna are well maintained, with a gain of ∼−28 dB. The proposed technology offers the possibility of realizing antenna-in-package (AiP) design on a flexible substrate with 2.5D integration circuits allowing for the high-speed communication at the 6G regime.

## Conclusions

In conclusion, we have developed a flexible nanoscale nonvolatile RF switch for 6G data communication. This switch combines memristor and RF functionalities, demonstrating low insertion loss and high isolation with a cutoff frequency up to 840 GHz. It has an on-state resistance of less than 50 Ω and an insertion loss of 2 dB, presenting stable performance after over 10^6^ switching cycles and 2500 bending cycles. Through the PSPI lithography process, we achieved 2.5D integration of antennas and RF switches, resulting in a return loss below 15 dB. The switch has a simple structure with high design flexibility readily for heterogeneous integration. We also demonstrate the high reliability which make it an ideal choice for next-generation communication technology, particularly in advancing packing in wearable electronics for 6G communication.

## Experimental Section

### Preparation of the Flexible Substrate

The entire device stack was developed on a flexible polyimide (PI) substrate. After cleaning with acetone and isopropanol, the PI substrate had been placed on a SiO_2_ substrate, and then a photosensitive polyimide (EverPI P09 PSPI) material was spin-coated onto the PI substrate. Subsequently, the coated substrate was placed in a vacuum and baked at a temperature of 180 °C for 2 h. PSPI films were spin-coated on Si substrates at speeds from 2900 to 3200 rpm (Figure S15). AFM showed low surface roughness (*R*_q_ = 0.3 nm) across all speeds, indicating minimal impact from the coating speed.

### Fabrication Process of Flexible RF Switches

After the substrate was prepared, the bottom electrode was defined through photolithography, and then 50 nm nickel was deposited by thermal evaporation, followed by a lift-off process to complete the fabrication of the bottom electrode. A 5 nm HfO_2_ insulation layer was grown using atomic layer deposition (ALD). The device active area of the HfO_2_ layer was defined using buffered oxide etchant (BOE) and photolithography. The antenna layers, including the antenna on the top and the common ground at the bottom of PSPI, were formed by depositing 50 nm nickel using photolithography and thermal evaporation. Each layer’s pattern was defined using self-aligned photolithography processes with masks specifically designed for high-frequency measurements. Atomic force microscopy images were collected by Bruker Dimension Icon (tip curvature radius <7 nm). SEM images were collected by Hitachi SU-8010 instrument with beam energy of 10–15 kV.

### Memristor Measurements

The devices were measured using a Keysight semiconductor parameter analyzer (B2902b) under ambient conditions. Pulse switching measurements were performed using Keysight waveform generator/fast measurement unit (WGFMU) module (B1500A with B1530).

### RF Measurements

The performance of the RF switch was characterized using the vector network analyzer (Keysight PNA-X network analyzer N5242B coupled with a Keysight N5293AX03 broadband frequency extender), covering the frequency range from 100 MHz to 110 GHz, using a RF probe for 110 GHz (MPI T110A). The *S*-parameter measurements used an input power of −5 dBm. Prior to device measurements, Short-Open-Load-Through (SOLT) calibration was performed using a calibration substrate (MPI AC-2), as shown in Figure S16. For power handling tests, additional equipment included a Keithley 2400 Standard Series SMU as the DC source and measurement unit, a VDI Model WR9.0SGX-M as the mini signal generator, and a VDI Erickson PM5B as the high-frequency power meter for millimeter-wave measurements at 94 GHz. Given the current measurement range capabilities of the equipment, the data obtained represent the best achievable measurements under these conditions. Figure S17 shows the *S*-parameter of the systems with and without the switch component as a comparison.

### Simulation of the 2.5D Integrated System

The gain and return loss of the antenna and the 2.5D integrated system were simulated using the high-frequency structure simulator (Ansys HFSS). Keysight Advanced Design System (ADS) was used for circuit simulation and for the calculation of the return loss.

## Data Availability

The data that support the findings of this study are available from the corresponding author upon reasonable request.

## Supporting Information Available

Experimental data, material characterization, and device design details; calculations of resistance and capacitance (*R* and *C*), *S*-parameter analysis, characteristic impedance, and loss tangent; high-frequency measurements provide insertion loss and isolation data across different substrates, along with statistical analysis of DC switching resistance; AFM and SEM confirm the uniformity and surface properties of the HfO_2_ film; de-embedding procedures, power handling, pulse measurement analysis, long-term stability, and interposer structure evaluations; and RF switch performance across various configurations (PDF)
