# Supplementary material for: HfO2 Memristor-Based Flexible Radio Frequency Switches
Source: ACS Nano. 2024 Dec 20;19(1):704–11. doi: 10.1021/acsnano.4c11846 (PMC11752509; doi:10.1021/acsnano.4c11846)
Supplement: Supplementary file 1 — nn4c11846_si_001.pdf [file nn4c11846_si_001.pdf]

Supplementary Information

HfO_2 Memristor-Based Flexible Radio-Frequency Switches

Shih-Chieh Chen[1,2], Yu-Tao Yang[3], Yun-Chien Tseng[1], Kun-Dong Chiou[1], Po-Wei Huang[1], Jia-Hao Chih[1], Hsien-Yang Liu[1], Tsung-Te Chou[4], Yang-Yu Jhang[4], Chien-Wei Chen[4], Chun-Hsiao Kuan[5], E Ming Ho[6], Chao-Hsin Chien[1], Chien-Nan Kuo[1], Yu-Ting Cheng[1]*, Der-Hsien Lien[1,2]**

[1]Institute of Electronics, National Yang Ming Chiao Tung University, Hsinchu, Taiwan

[2]Institute of Pioneer Semiconductor Innovation, National Yang Ming Chiao Tung University, Hsinchu, Taiwan

[3]Strategic Technology Exploration Platform, MediaTek, San Jose, USA

[4]Taiwan Instrument Research Institute, National Applied Research Laboratories, Hsinchu, Taiwan

[5]Department of Applied Chemistry and Institute of Molecular Science, National Yang Ming Chiao Tung University, Hsinchu, Taiwan

[6]Chang Chun Plastics Co., Ltd. Hsinchu Factory, Hsinchu, Taiwan

*Address correspondence to dhlien@nycu.edu.tw, ytcheng@g2.nctu.edu.tw, cnkuo@nycu.edu.tw

This supplementary information file includes:

Supplementary Fig. 1-17

Table S1

Detailed calculations of *R* & *C*

Analysis of *S*-parameters, characteristic impedance, and loss tangent

Supplementary references 1–14

Detailed calculations of *R* & *C*

In microwave engineering, the *S*-parameters measured by a Vector Network Analyzer (VNA) are typically provided in terms of magnitude and phase. To analyze these parameters, they must be converted into impedance (*Z*) and subsequently into admittance (*Y*).

1. Steps for Converting *S*-parameters to Impedance and Admittance

Given an *S*-parameter of the form:

$$S = \mathrm{M} \times (\cos(\theta) + j \cdot \sin\theta)) \quad (1)$$

Where M is the magnitude (linear unit) and θ is the phase angle (radians). To convert dB values to linear magnitude and phase angle, linear magnitude(M)= $10^{\frac{dB\ value}{20}}$, radians($\theta$)=degrees×$\frac{\pi}{180}$, real part (Re)**=** M×cos(θ) and imaginary part (Im)**=** M×sin(θ). These conversions help in translating the *S*-parameters into a format suitable for further analysis.

2. Impedance and Admittance Conversion Formulas

For the *S*-parameter matrix:

$$S = \begin{pmatrix} S_{11} & S_{12} \\ S_{21} & S_{22} \end{pmatrix} \quad (2)$$

The admittance matrix *Y* can be directly calculated from the *S*-parameters using the following formula, as detailed in *Microwave Engineering*:[1,2]

$$Y = \frac{1}{Z_0}\begin{pmatrix} (1+S_{11})(1-S_{22}) + S_{12}S_{21} & -2S_{12} \\ -2S_{21} & (1-S_{11})(1+S_{22}) + S_{12}S_{21} \end{pmatrix} \quad (3)$$

Where Z_0 is the characteristic impedance of the network, typically 50 ohms. This formula provides a straightforward method to convert *S*-parameters to the corresponding *Y*-parameters without the need for intermediate steps. Expanding this, the individual elements of the admittance matrix *Y* are given by:

$$Y_{11} = \frac{1}{Z_0} \times \frac{(1 + S_{11})(1 - S_{22}) + S_{12}S_{21}}{\Delta S} \quad (4a)$$

$$Y_{12} = \frac{1}{Z_0} \times \frac{-2S_{12}}{\Delta S} \quad (4b)$$

$$Y_{21} = \frac{1}{Z_0} \times \frac{-2S_{21}}{\Delta S} \quad (4c)$$

$$Y_{22} = \frac{1}{Z_0} \times \frac{(1 - S_{11})(1 + S_{22}) + S_{12}S_{21}}{\Delta S} \quad (4d)$$

These formulas provide a straightforward method to convert *S*-parameters to the corresponding Y-parameters, accounting for both the direct and cross-port interactions in a two-port network.

3. Radio frequency (RF) Switch State Analysis

The ON and OFF states of the RF switch can be analyzed using the admittance parameters. OFF state capacitance *C* can be calculated as:

$$C = \frac{Im(Y_{12})}{2\pi f} \quad (5)$$

ON state resistance *R* can be calculated as:

$$R = \frac{1}{\mathrm{Re}(Y_{12})} \quad (6)$$

Analysis of *S*-parameters, characteristic impedance, and loss tangent

In RF circuit design, *S*-parameters are crucial for describing the behavior of RF networks, particularly in terms of signal reflection and transmission. Specifically, S_{11} represents the reflection coefficient, while S_{21} represents the transmission coefficient. These parameters are mathematically defined as:[3]

$$S_{11} = \frac{(Z^2 - Z_0^2)\sinh(\gamma l)}{2ZZ_0\cosh(\gamma l) + (Z^2 + Z_0^2)\sinh(\gamma l)} \quad (1)$$

$$S_{21} = \frac{2ZZ_0}{2ZZ_0\cosh(\gamma l) + (Z^2 + Z_0^2)\sinh(\gamma l)} \quad (2)$$

In these equations, Z refers to the impedance of the transmission line, while Z_0 denotes the characteristic impedance. γ represents the propagation constant, and l stands for the length of the transmission line.

The relationship between *S*-parameters and the characteristic impedance can be derived using:

$$\left(\frac{Z}{Z_0}\right)^2 = \frac{1 + S_{11} + S_{21}}{1 - S_{11} - S_{21}} \times \frac{1 + S_{11} - S_{21}}{1 - S_{11} + S_{21}} \quad (3)$$

The characteristic impedance Z_0 depends on the physical dimensions of the transmission line, such as the width of microstrip line W, the thickness T of the conductor, the height H of the substrate, and the effective dielectric constant ε_e. It is expressed as:[4]

$$Z_0 = \frac{1}{\frac{W_{eff}}{H} + 2.42 - 0.44\left(\frac{H}{W_{eff}}\right) + \left(1 - \frac{H}{W_{eff}}\right)^6} \times \frac{120\pi}{\sqrt{\varepsilon_e}} \quad (4)$$

Where $\varepsilon_e = \frac{\varepsilon_r + 1}{2} + \frac{\varepsilon_r - 1}{2}\left(1 + \frac{10H}{W}\right)^{-\frac{1}{2}}$. In this context, ε_r is the relative dielectric constant of the substrate material, and W_{eff} is the effective width of the transmission line. This effective width must be adjusted when the trace thickness T is non-

negligible, particularly when T/H > 0.005. The actual effective width is given by:

$$W_{eff} = W + \left(\frac{T}{\pi}\right)\left(\ln\frac{2H}{T} + 1\right) \quad (5)$$

This correction accounts for the impact of the conductor's thickness T on the characteristic impedance.

Additionally, the loss tangent $\tan\delta$ is crucial for understanding dielectric losses. The total loss α in a microstrip line is the sum of the conductor loss α_c and the dielectric loss α_d, where $\alpha = \alpha_c + \alpha_d = -\frac{1}{L}\ln(\sqrt{|S_{11}|^2 + |S_{21}|^2})$.

Conductor loss α_c is expressed as:[5]

$$\alpha_c = \frac{10R_s}{\pi \ln 10} \frac{\left(\frac{8H}{W} - \frac{W}{4H}\right)\left(1 + \frac{H}{W} + \frac{H}{W}\left(\frac{1}{\pi}\ln\frac{2H}{T}\right)\right)}{HZ_0 e^{\left(\frac{Z_0}{60}\right)}} \quad (6)$$

The loss tangent $\tan\delta$ is related to the dielectric loss by:

$$\tan\delta = \frac{\sigma}{\omega\varepsilon} = \frac{2\alpha_d}{\beta} \quad (7)$$

In this context, β represents the phase constant, which is a component of the propagation constant γ. The phase constant β describes the rate at which the phase of the electromagnetic wave changes along the transmission line, measured in radians per meter (rad/m). This parameter is crucial for understanding how the dielectric loss affects the phase and energy dissipation as the signal propagates through the material. Additionally, σ is the conductivity, ω is the angular frequency, and ε is the permittivity. These equations are essential for analyzing the interaction between *S*-parameters, characteristic impedance, and loss tangent, and are critical for optimizing RF switch performance in high-frequency and flexible applications.

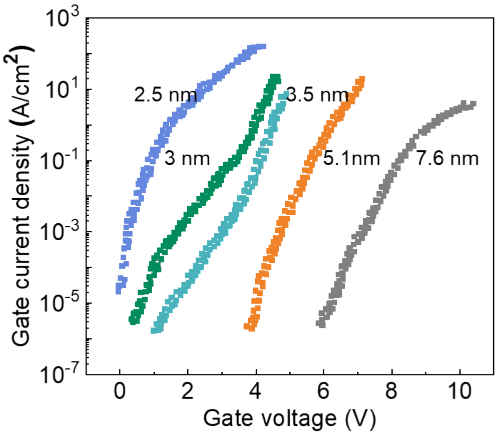

Fig. S1. Optimization of Hafnium dioxide (HfO_2) Thickness for Gate Leakage Control and RF Switch Performance. To minimize gate leakage while ensuring sufficient isolation and controlling insertion loss, the HfO_2 active layer is optimized at 5 nm thickness. Thinner layers increase leakage, as shown in the gate current density plot.[6]

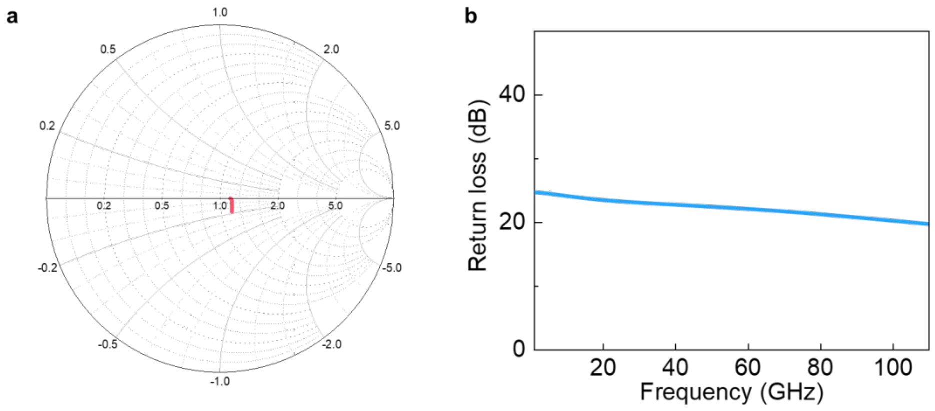

Fig. S2. Impedance matching of the RF switch designed. **(a)** Smith chart showing the impedance matching performance of the RF switch based on the coplanar waveguide (CPW) structure, with return loss data across the entire frequency range. **(b)** Return loss greater than 20 dB, indicating stable matching and low reflection characteristics for the RF switch in this configuration.

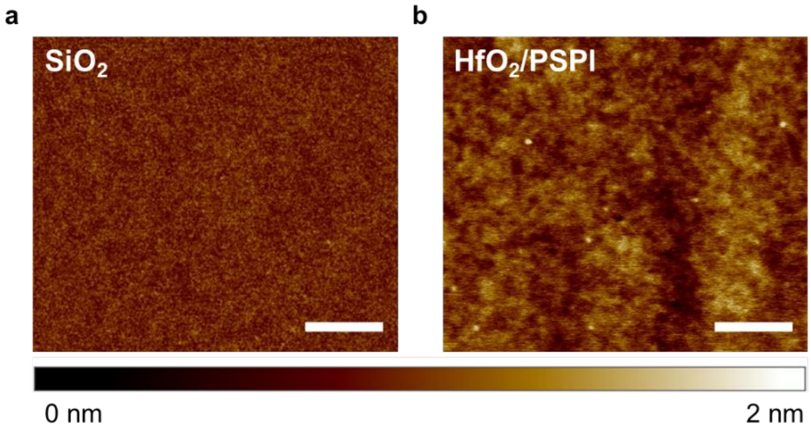

Fig. S3. Atomic force microscope (AFM) images of roughness. **(a)** The AFM images demonstrate that the SiO_2 surface with an Rq of approximately 0.2 nm. **(b)** The data show that the roughness of HfO_2 deposited on PSPI substrates is approximately 0.2 nm, similar to that of HfO_2 grown on Nickel films, indicating that the film quality of HfO_2 within the device area is reliable.

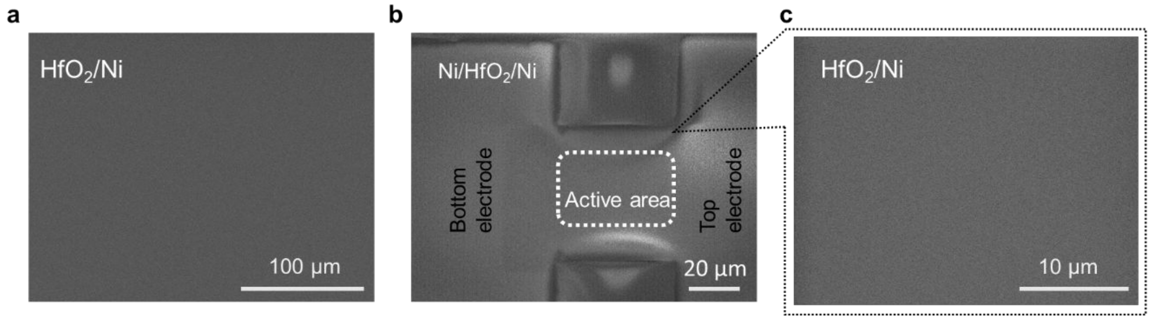

Fig. S4. SEM images of the HfO_2 surface at different magnifications. **(a)** A large-area SEM scan of the HfO_2 layer on nickel metal, with a scale bar of 100 μm. **(b)** The SEM image shows the active area of the Ni/HfO_2/Ni stack, where the HfO_2 layer is sandwiched between the top electrode (TE) and bottom electrode (BE), with a scale bar of 20 μm. **(c)** SEM scan of the HfO_2 layer in the active region, with a scale bar of 10 μm, demonstrating a smooth and uniform surface without noticeable grains or defects.

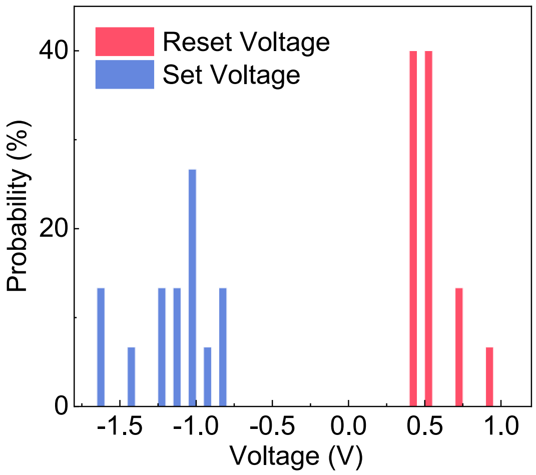

Fig. S5. Low voltages probability distribution. The probability distribution associated with the switching events during the set and reset operations is shown, with the set operation centered around -1 V and the reset operation centered around 0.5 V.

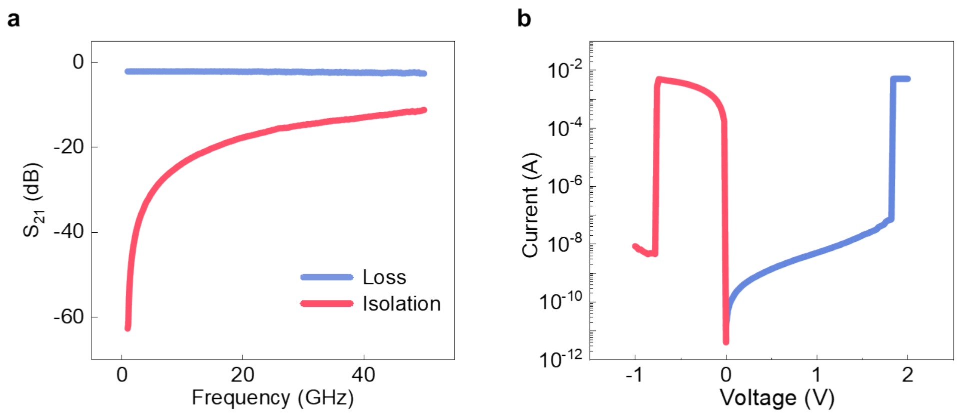

Fig. S6. High frequency and memristor performance of quartz substrate Al_2O_3 RF switch. **(a)** The *S*-parameter performance of the RF switch on a quartz substrate. The blue line representing the insertion loss and the red line representing isolation across a range of frequencies up to 50 GHz. The loss remains low, while the isolation increases with frequency, indicating effective signal separation. **(b)** The current-voltage (I-V) characteristics of the memristor-based RF switch. The blue curve indicates that the device begins to conduct at nearly 2 volts, while the red curve shows the device turns off at around -0.5 volts.

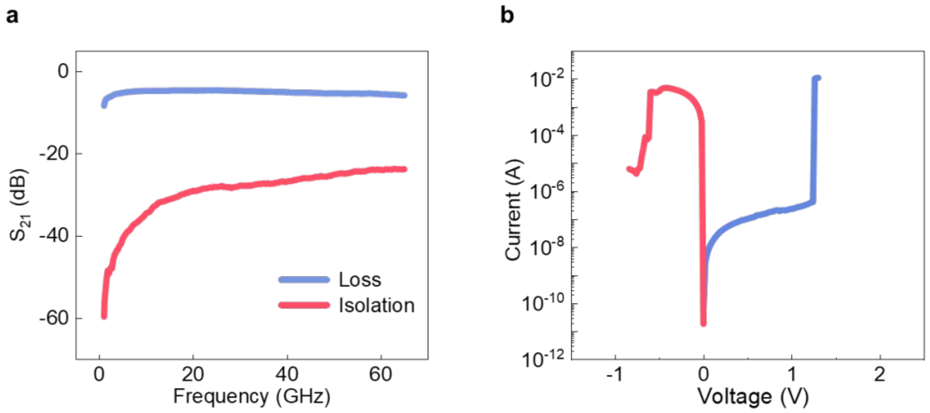

Fig. S7. High frequency and memristor performance of SiO_2/PSPI substrate Al_2O_3 RF switch with 20 μm² ingle device area. **(a)** The high-frequency *S*-parameter performance of an Al_2O_3 RF switch on a SiO_2/PSPI substrate with a single device area of 20 μm². The blue line represents insertion loss, and the red line represents isolation. As frequency increases, loss remains stable, while isolation improves. At 67 GHz, the insertion loss is 4 dB, and isolation is 23 dB. **(b)** The I-V characteristics of the memristor-based RF switch. The blue curve indicates that the device begins to conduct at nearly 1.3 volts, while the red curve shows the device turns off at around -0.5 volts.

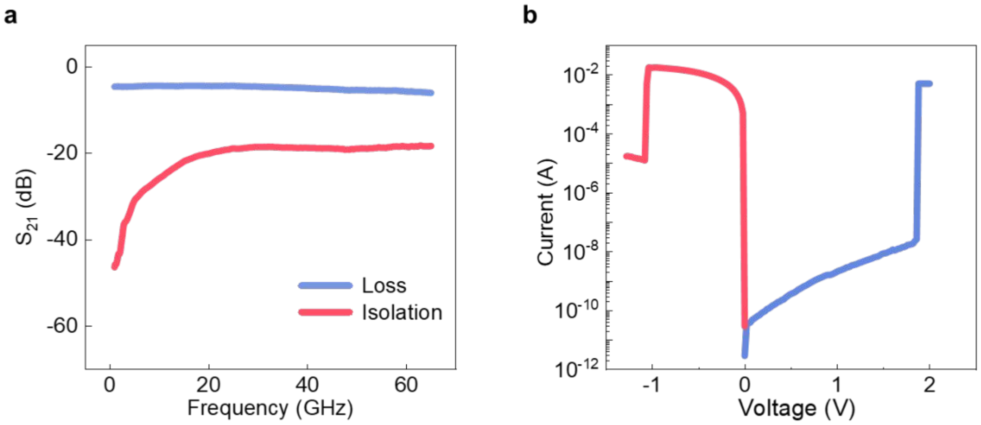

Fig. S8. High frequency and memristor performance of SiO_2/PSPI substrate Al_2O_3 RF switch with 10 μm² ingle device area. **(a)** The high-frequency *S*-parameter performance of an Al_2O_3 RF switch on a SiO_2/PSPI substrate with a single device area of 10 μm². At 67 GHz, the insertion loss is 4 dB, and isolation is 20 dB. **(b)** The I-V characteristics of the memristor-based RF switch. The blue curve indicates that the device begins to conduct at nearly 2 volts, while the red curve shows the device turns off at around -1 volts.

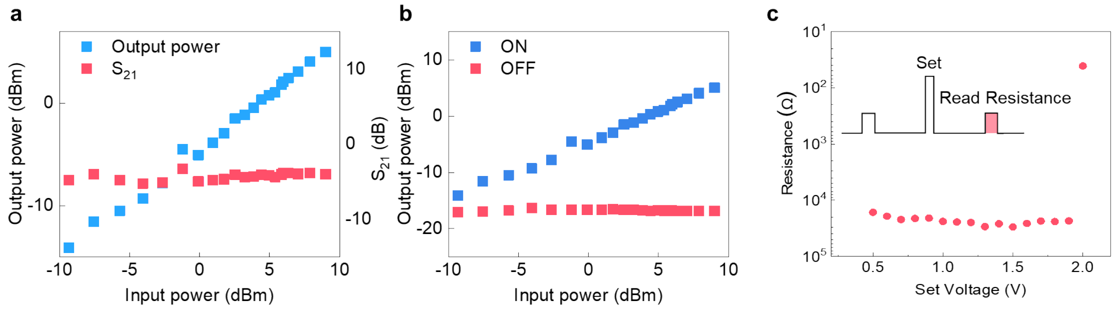

Fig. S9. Power handling characteristics of the RF switch, measured at a frequency of 94 GHz. **(a)** Power handling characteristics of the RF switch in the ON state. The insertion loss shows a slight variation with increasing input power but remains generally stable. **(b)** Power handling characteristics of the RF switch in both ON and OFF states, with the OFF state showing a flat output power curve, indicating effective isolation of the input signal. **(c)** Pulse test results at different input voltages, showing no state change until the input voltage reaches 2 V, confirming no self-switching behavior at an input power close to 10 dBm with a peak voltage (Vp) of 1 V.

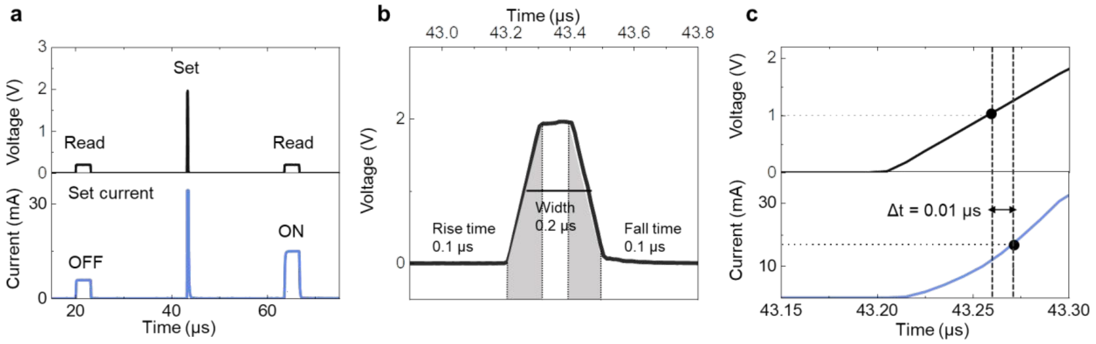

Fig. S10. Analysis of pulse parameters and switching characteristics of the device. **(a)** Set pulse voltage and current. **(b)** Voltage waveform showing rise/fall times and Full Width at Half Maximum (FWHM) positions. **(c)** A delay of approximately 0.01 µs between the FWHM positions of the voltage and current waveforms.

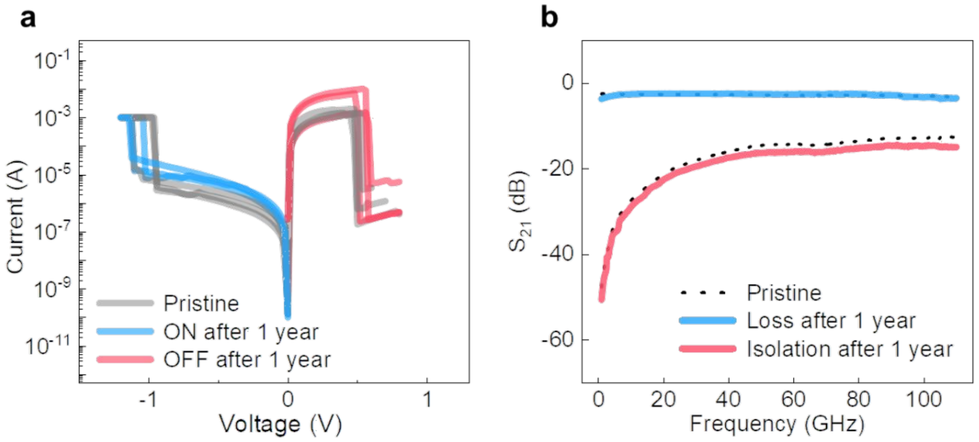

Fig. S11. One-Year Device Performance**. (a)** I-V characteristics of the device before and after one year of storage, showing no significant changes in the ON and OFF states. **(b)** S_{21} performance of the RF switch, demonstrating that both insertion loss and isolation remain stable after one year, confirming long-term reliability.

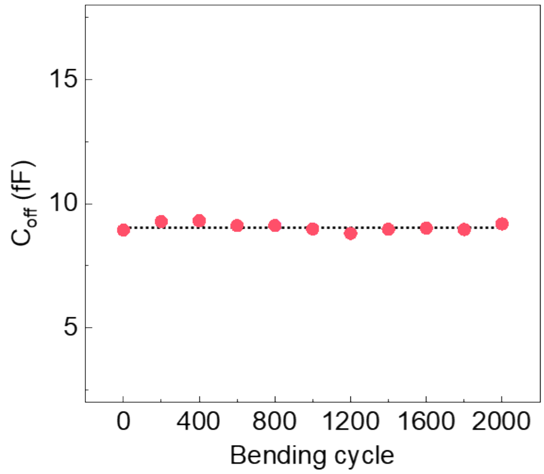

Fig. S12. OFF-state capacitance. Changes in OFF-state capacitance under different bending cycles, showing only slight fluctuations

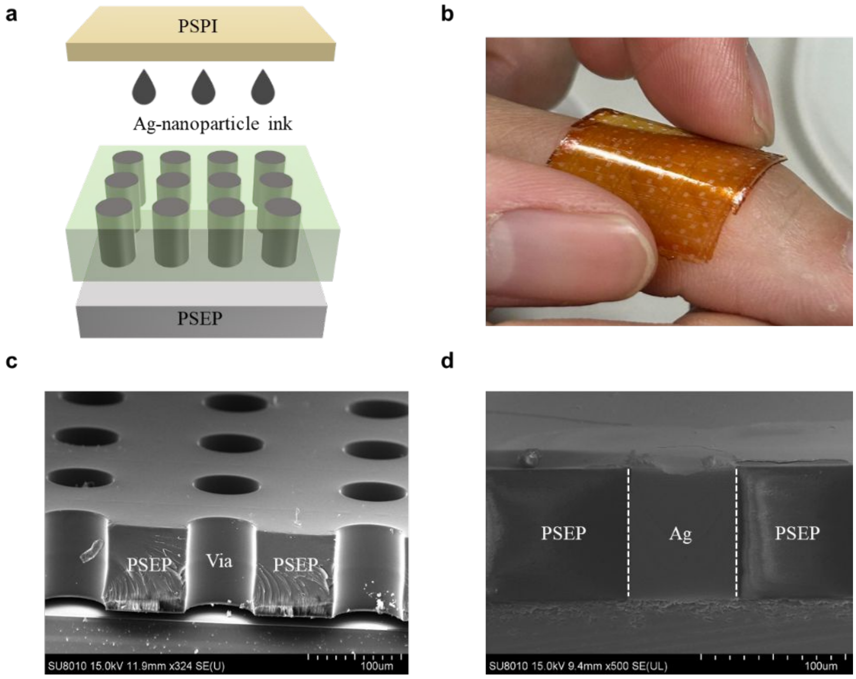

Fig. S13. Fabrication and SEM analysis of photosensitive epoxy (PSEP) based Flexible interposer with inkjet-printed silver via filling. **(a)** Schematic illustration of the PSEP interposer fabrication process, where vias are created and subsequently filled with silver ink using inkjet printing technology. **(b)** Demonstration of the flexibility of the PSEP material, showing it wrapped around a finger, highlighting its suitability as a flexible substrate material. **(c)** Scanning electron microscope (SEM) image shows the cross-section of the PSEP interposer, the via profile after the photolithography process. **(d)** SEM shows a via filled with silver (Ag) in the PSEP interposer, indicating the quality of the via fill and the integrity of the PSEP as a flexible substrate material.

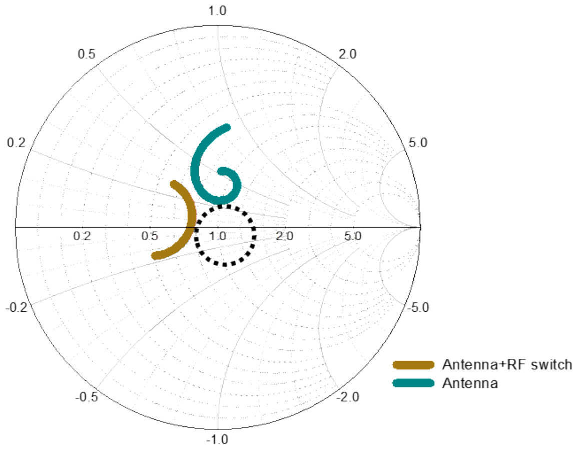

Fig. S14. Smith chart illustrating the impedance matching characteristics. The S_{11} of the antenna and the antenna combined with the RF switch both fall on the same impedance circle, indicating impedance matching.

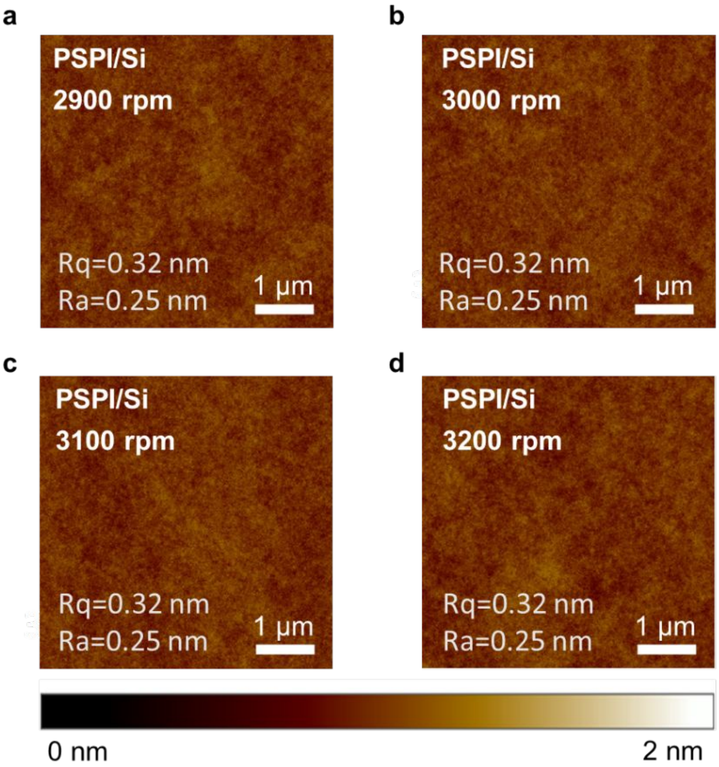

Fig. S15. AFM images of PSPI substrate roughness. AFM images indicate the surface morphology of PSPI on Si substrates at various spin-coating speeds (2900 to 3200 rpm). Despite the variation in coating speeds, the surface roughness parameters Rq is 0.3 nm and Ra is 0.2 nm. This indicates that the coating speed does not significantly affect the surface roughness of the PSPI films, maintaining a consistently smooth surface across all tested conditions.

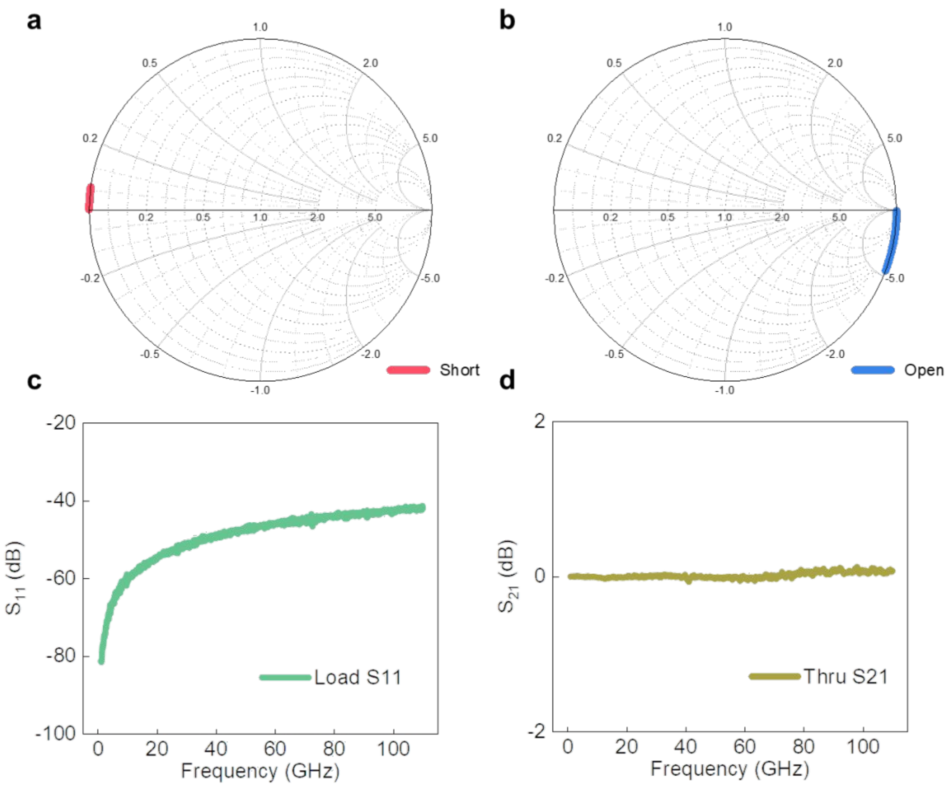

Fig. S16. Short-Open-Load-Through (SOLT) calibration. **(a,b)** Show the Smith charts for Short and Open. **(c)** Shows the S_{11} characteristics of the Load. **(d)** Shows the S_{21} characteristics of the Thru. The results indicate that the SOLT calibration values meet the standards and align with expectations.

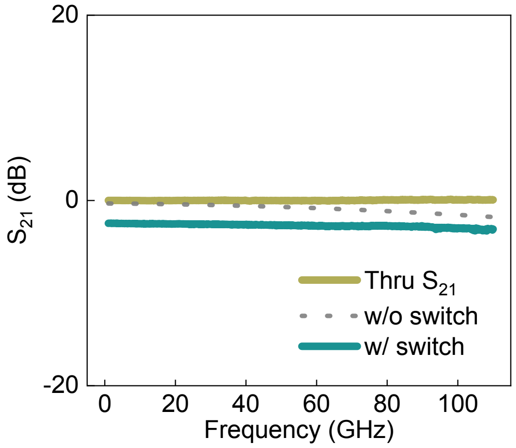

Fig. S17. Without switch result. The intrinsic RF transmission characteristics of the switch are extracted by removing the influence of the metal pads from the measured *S*-parameters.

Table S1. RF switch technologies comparison table

Reference	Device technology	Flexible	Non-volatility	Integration capability	Cutoff frequency	Maximum frequency S_{21} insertion loss/isolation	Maximum Operating Frequency	Maximum pulse time	Operating voltage (V)	Bending cycle	Bending operating frequency	Switching time
**This work**	**Memristor switch**	**Yes**	**Yes**	**2.5D interposer**	**840 GHz**	**2/13 dB**	**110 GHz**	**1 M**	**0.5 - 1**	**2600**	**67 GHz**	**< 10 ns**
Shi et al., npj Flex Electron., 2022[7]	MEMS switch	Yes	No	Not reported	Not reported	0.06/45 dB	20 GHz	Not reported	~60	Not reported	40 GHz	1-300 μs
Yang et al., Adv. Mater., 2019[8]	MIT	Yes	No	Not reported	Not reported	4/13 dB	40 GHz	Not reported	~4	Not reported	Not reported	0.4 μs
Kim et al., Nat. Elect., 2020[9]	hBN switch	No	Yes	Not reported	129 THz	0.27/35 dB	67 GHz	Not reported	~1- 3	Not reported	Not reported	< 10 ns
Kim et al., Nat. Elect., 2022[10]	MoS_2 switch	No	Yes	Not reported	11 THz	1/20 dB	480 GHz	Not reported	0.5 - 1.5	Not reported	Not reported	500 ps
Leon et al., IEEE Trans. Microw. Theory Tech, 2019[11]	PCM switch	No	Yes	Not reported	21 THz	0.15/14 dB	40 GHz	Not reported	1.1	Not reported	Not reported	100 ns
Pi et al., Nat. Comms., 2015[12]	CBRAM switch	No	Yes	Not reported	35 THz	0.3/40 dB	40 GHz	Not reported	3	Not reported	Not reported	1-100 ns
Patel et al., IEEE Trans. Microw. Theory Tech, 2012[13]	MEMS switch	No	No	Not reported	12.4 THz	0.5/20 dB	40 GHz	Not reported	80 - 90	Not reported	Not reported	1-300 μs
Field et al., Proc. SPIE, 2015[14]	IMT	No	No	Not reported	45 THz	0.5/18 dB	110 GHz	Not reported	~2	Not reported	Not reported	2 μs

REFERENCES

(1) Pozar, D. M. *Microwave engineering: theory and techniques*; John wiley & sons, **2021**.

(2) Rizzi, P. A. *Microwave engineering: passive circuits*; Prentice Hall New Jersey, **1988**.

(3) Yang, L.-l.; Sun, L.; Shi, Q.; Sun, H.-y. Extraction of dielectric constant based on S-parameter inversion method. In *2011 3rd International Conference on Computer Research and Development*, **2011**; IEEE: Vol. 3, pp 353-356.

(4) Hammerstad, E. O. Equations for microstrip circuit design. In *1975 5th European Microwave conference*, **1975**; IEEE: pp 268-272.

(5) Wu, S.-M.; Lai, C.-C.; Cheng, H.-H.; Tai, Y.-C.; Wang, C.-C. Frequency dielectric constant and loss tangent extracting of organic material using multi-length microstrip. In *2008 International Conference on Electronic Packaging Technology & High Density Packaging*, **2008**; IEEE: pp 1-4.

(6) Hu, C. Gate oxide scaling limits and projection. In *International Electron Devices Meeting. Technical Digest*, **1996**; IEEE: pp 319-322.

(7) Shi, Y.; Zhou, C.; Cao, Z.; He, Y.; Guo, J.; Li, C.; Wu, Q.; Liang, K.; Li, Y.; Lin, Y. Flexible radio-frequency micro electro-mechanical switch towards the applications of satellite communications. *npj Flexible Electron.* **2022**, *6* (1), 80.

(8) Yang, S.; Vaseem, M.; Shamim, A. Fully inkjet-printed VO2-based radio-frequency switches for flexible reconfigurable components. *Adv. Mater. Technol.* **2019**, *4* (1), 1800276.

(9) Kim, M.; Pallecchi, E.; Ge, R.; Wu, X.; Ducournau, G.; Lee, J. C.; Happy, H.; Akinwande, D. Analogue switches made from boron nitride monolayers for application in 5G and terahertz communication systems. *Nat. Electron.* **2020**, *3* (8), 479-485.

(10) Kim, M.; Ducournau, G.; Skrzypczak, S.; Yang, S. J.; Szriftgiser, P.; Wainstein, N.; Stern, K.; Happy, H.; Yalon, E.; Pallecchi, E. Monolayer molybdenum disulfide switches for 6G communication systems. *Nat. Electron.* **2022**, *5* (6), 367-373.

(11) Leon, A.; Reig, B.; Perret, E.; Podevin, F.; Saint-Patrice, D.; Puyal, V.; Lugo-Alvarez, J.; Ferrari, P. RF power-handling performance for direct actuation of germanium telluride switches. *IEEE Trans. Microwave Theory Tech.* **2019**, *68* (1), 60-73.

(12) Pi, S.; Ghadiri-Sadrabadi, M.; Bardin, J. C.; Xia, Q. Nanoscale memristive radiofrequency switches. *Nat. Commun.* **2015**, *6* (1), 7519.

(13) Patel, C. D.; Rebeiz, G. M. A high-reliability high-linearity high-power RF MEMS metal-contact switch for DC–40-GHz applications. *IEEE Trans. Microwave Theory Tech.* **2012**, *60* (10), 3096-3112.
(14) Field, M.; Hillman, C.; Stupar, P.; Hacker, J.; Griffith, Z.; Lee, K.-J. Vanadium dioxide phase change switches. In *Open Architecture/Open Business Model Net-Centric Systems and Defense Transformation 2015*, **2015**; SPIE: Vol. 9479, pp 31-38.
